# Supplementary material for: The experience of a program combining two complementary therapies for women with breast cancer: An IPSE qualitative study
Source: PLoS One. 2023 Aug 17;18(8):e0285617. doi: 10.1371/journal.pone.0285617 (PMC10434849; doi:10.1371/journal.pone.0285617)
Supplement: S1 Table — (DOCX) [file pone.0285617.s001.docx]

**Table S1. Participants’ characteristics**

| **Woman** | **Age range** | **Treatments received** | **Program of complementary therapies (MBSR and stationary biking)** | **Other complementary therapies** |
| --- | --- | --- | --- | --- |
| 1 | 50-60 | S + CT + RT + HT | MBSR, stationary biking |  |
| 2 | 50-60 | S + CT + RT + HT | MBSR | Dietetics, Reconstructive/plastic surgical care, Psychological therapy |
| 3 | 50-60 | S + CT + RT + HT | MBSR, Stationary biking | Reconstructive/plastic surgical care,Yoga, Homeopathy |
| 4 | 50-60 | S + CT + RT + HT | MBSR, Stationary biking | Tai Chi, Qi Qong, Homeopathy |
| 5 | 50-60 | S + CT + RT + HT | MBSR, Stationary biking |  |
| 6 | 40-50 | S + CT + RT | MBSR, Stationary biking |  |
| 7 | 50-60 | S + CT | MBSR, Stationary biking | Reiki, Massage, Homeopathy, Psychological therapy, Physical therapy |
| 8 | 60-70 | S + CT + HT | MBSR, Stationary biking | Rowing, Nordic walking, Yoga, Qi Qong, Reflexology, Thermal cure, Psychological therapy, Physical therapy |
| 9 | 50-60 | S + CT + RT+ HT | MBSR, Stationary biking | Psychological therapy, Physical therapy |
| 10 | 60-70 | S + CT + RT + HT | Stationary biking | Homeopathy care, Physical therapy |
| 11 | 50-60 | S + CT + RT + HT | Stationary biking | Reiki, Reflexology, Massage, Homeopathic care, Psychological therapy |
| 12 | 50-60 | S+ CT + RT + HT | MBSR, Stationary biking | Nordic walking, Homeopathic care, Psychological therapy |
| 13 | 50-60 | S + CT + HT | MBSR, Stationary biking | Yoga |
| 14 | 50-60 | S + CT + RT+ HT | Stationary biking | Nordic walking, Physical therapy |
| 15 | 60-70 | S + CT + HT | MBSR, Stationary biking | Yoga, Rowing, Homeopathic care, Psychological therapy, Physical therapy |
| 16 | 50-60 | S + CT + RT+ HT | Stationary biking | Thermal cure |
| 17 | 50-60 | S + CT + RT | MBSR | Qi gong, Psychological therapy, Physical therapy |
| 18 | 40-50 | S + CT + RT+ HT | Stationary biking | Reconstructive/plastic surgical care, Physical therapy |
| 19 | 50-60 | S + CT + RT | MBSR, Stationary biking |  |
| 20 | 40-50 | S +CT + RT | MBSR, Stationary biking | Physical therapy, Psychological therapy |
| 21 | 50-60 | S + CT + RT + HT | NA |  |
| 22 | 60-70 | CT + S +RT +HT | MBSR, Stationary biking |  |
| 23 | 40-50 | S + CT + RT + HT | MBSR, Stationary biking | Boxing, Nordic walking |
| 24 | 60-70 | CT + S + RT | MBSR | Joined the League against cancer, Psychological therapy |
| 25 | 40-50 | CT + S + RT | MBSR, Stationary biking | Yoga |
| 26 | 50-60 | S + CT + RT | MBSR, Stationary biking | Reconstructive/plastic surgical care |
| 27 | 50-60 | CT + S + RT + CT | MBSR, Stationary biking | Yoga, Psychological therapy |
| 28 | 40-50 | S + CT | MBSR, Stationary biking | Psychiatric care, Support group |
| 29 | 50-60 | CT + S + RT | MBSR, Stationary biking | Gymnastics |

S: Surgery n=29; CT: Chemotherapy n=29; HT: Hormone therapy n=18; MBSR: Mindfulness-based stress reduction; NA : Not available; RT: Radiation therapy
